# Identification of stromal proteins overexpressed in nodular sclerosis Hodgkin lymphoma

**DOI:** 10.1186/1477-5956-9-63

**Published:** 2011-10-05

**Authors:** Philippe Kischel, David Waltregny, Yannick Greffe, Gabriel Mazzucchelli, Edwin De Pauw, Laurence de Leval, Vincent Castronovo

**Affiliations:** 1 Metastasis Research Laboratory, GIGA Cancer, University of Liege, Bat. B23, CHU Sart Tilman Liège, B-4000 Liège, Belgium; 2 Laboratory of Mass Spectrometry, Department of Chemistry, University of Liege, Bat. B6c, B-4000 Liège, Belgium; 3 Department of Pathology, University Hospital of Liege, Bat. B23, CHU Sart Tilman Liège, B-4000 Liege, Belgium

**Keywords:** Biomarker discovery, Lymphoma, Mass spectrometry, Tumour targeting

## Abstract

Hodgkin lymphoma (HL) represents a category of lymphoid neoplasms with unique features, notably the usual scarcity of tumour cells in involved tissues. The most common subtype of classical HL, nodular sclerosis HL, characteristically comprises abundant fibrous tissue stroma. Little information is available about the protein composition of the stromal environment from HL. Moreover, the identification of valid protein targets, specifically and abundantly expressed in HL, would be of utmost importance for targeted therapies and imaging, yet the biomarkers must necessarily be accessible from the bloodstream. To characterize HL stroma and to identify potentially accessible proteins, we used a chemical proteomic approach, consisting in the labelling of accessible proteins and their subsequent purification and identification by mass spectrometry. We performed an analysis of potentially accessible proteins in lymph node biopsies from HL and reactive lymphoid tissues, and in total, more than 1400 proteins were identified in 7 samples. We have identified several extracellular matrix proteins overexpressed in HL, such as versican, fibulin-1, periostin, and other proteins such as S100-A8. These proteins were validated by immunohistochemistry on a larger series of biopsy samples, and bear the potential to become targets for antibody-based anti-cancer therapies.

## Background

Human malignant lymphomas encompass a large variety of lymphoid neoplasms, that broadly are classified as Hodgkin lymphomas (HL) or non-Hodgkin lymphomas (NHL). One of the hallmark features of classical HL is the relative scarcity of typically large or giant neoplastic cells (Reed-Sternberg cells and variants), dispersed within an abundant mixed reactive cellular infiltrate and associated with a variably prominent stroma [1].

Importantly, bidirectional interactions involving soluble factors and membrane-bound receptors are known to take place between Reed-Sternberg cells and the various types of surrounding non-neoplastic cells, and the reactive component of HL tissues is suspected to play a major role in sustaining tumour development and progression [2]. In addition, the stromal compartment of many neoplastic tissues is fundamentally different from that of the corresponding normal tissue, and is suspected to promote cancer progression [3]. In this respect, little is known about the composition of the extracellular matrix (ECM) of HL. A comprehensive determination of the composition of the stromal compartment in HL appears therefore essential for a better understanding of the disease, and could lead to the discovery of new diagnostic and therapeutic markers allowing earlier detection and treatment of relapsed and refractory HL. Recurrent and refractory HL remain indeed an unmet challenge, and new strategies to improve outcome and reduce complications after standard therapy are required.

The use of gene microarrays can provide valuable information about the regulation of gene expression [4]. However, it has become increasingly evident over the past decade that mRNA quantitation does not always reflect corresponding protein levels [5,6]. In this context, proteome analyses provide a direct snapshot of the structural and functional framework of cellular life, and remain the best way to characterize a cellular environment and to identify new candidate biomarkers. So far, methods used to identify protein markers in lymphoma include enzyme-linked immunosorbent assays (ELISA), immunohistochemistry (IHC) on tissue microarrays, and mass-spectrometry (MS). However, proteomic analyses of human lymphoma are still very limited, and most of the studies were conducted on cell lines [7-9]. Only scarce data from high-throughput proteomic data are currently available for human nodular sclerosis HL tissues [10], while data are available for human NHL tissues, including mantle cell lymphoma [11-13], diffuse large B-cell lymphoma [14], small lymphocytic lymphoma and marginal zone lymphoma [13].

Herein, we performed an analysis of potentially accessible proteins in human HL (n = 4), in comparison to benign lymphoid tissue (reactive lymphoid hyperplasia, RLH, n = 3). To this end, we used a recently described chemical proteomic technology [15], which permits the identification of accessible antigens through biotinylation of exposed primary amines. This technique allowed us to analyse, among others, the ECM proteins of our samples. Overall, over 1430 proteins were identified in all HL or RLH tissues. We found differentially expressed ECM proteins in HL, such as versican, fibulin-1, periostin, and other proteins such as S100-A8. These proteins were validated by immunohistochemistry and may become potential targets for either diagnostic or therapeutic purposes.

## Results

### Validation of the biotinylation step

**Figure 1:**
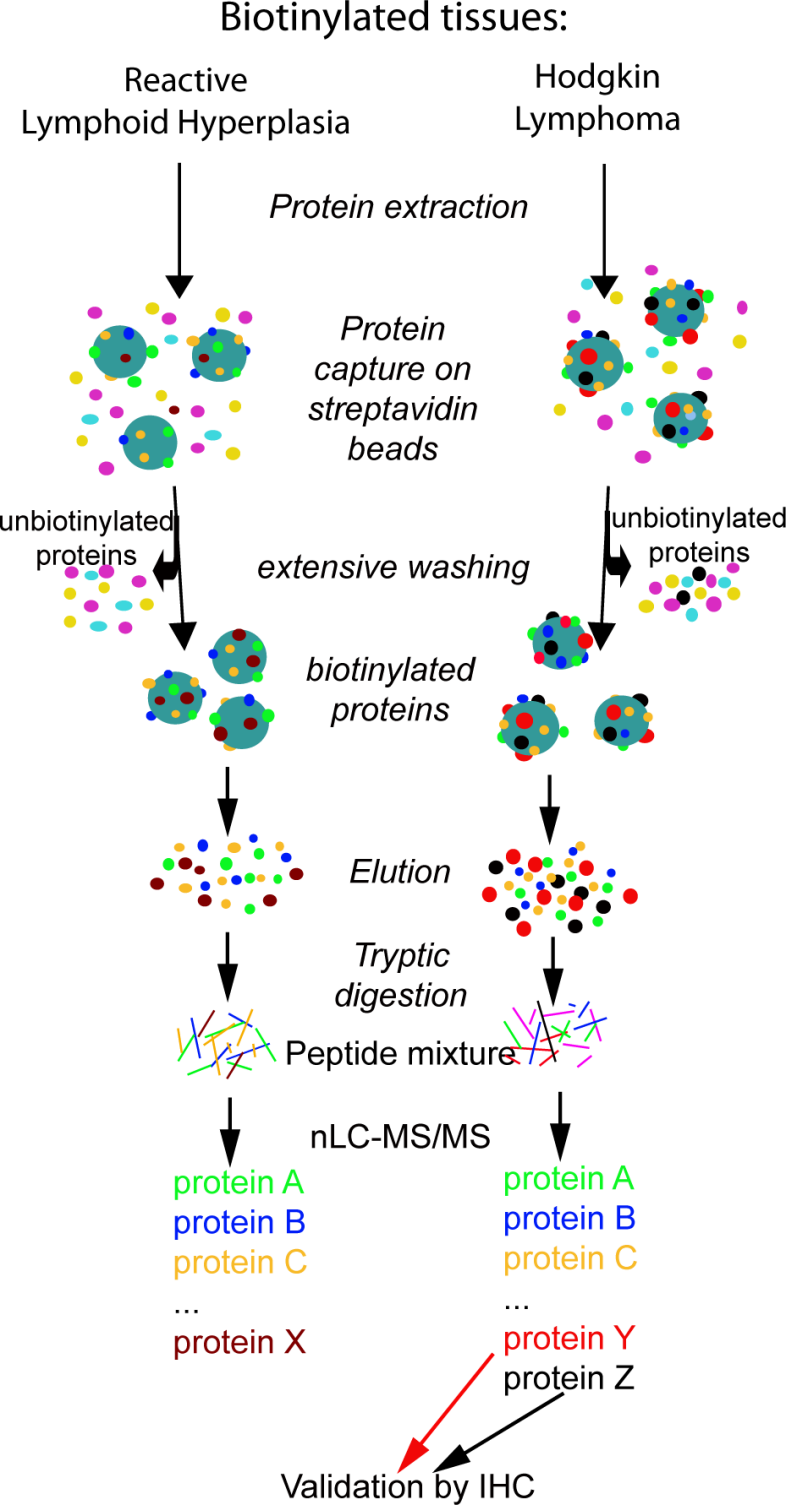
**Schematic description of the method used in this study for the identification of accessible proteins in Hodgkin lymphoma and reactive lymph nodes**. *Ex vivo *biotinylation of human tissue samples was carried out by incubating fresh tissues in a solution containing reactive ester derivatives of biotin. Proteins from each tissue sample were extracted. Biotinylated proteins were then captured on streptavidin resin, eluted thanks to the disulfide bond between the lysines and the biotin, alkylated and submitted to proteolytic digestion. Biotinylated proteins were sequenced by shotgun mass spectrometry using nLC-ESI MS/MS. Identified proteins differentially expressed in the tissue samples were further validated by immunohistochemical analysis.

**Figure 2:**
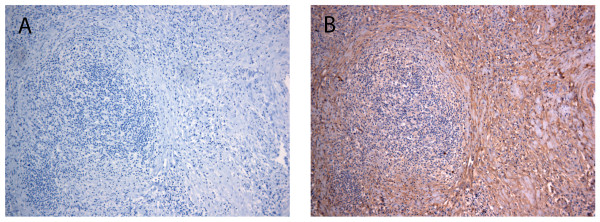
**Evaluation of the biotinylation efficiency**. Thin slices of lymph nodes (in this case HL) were soaked in the biotinylation solution for 20 minutes at 37°C. Extent of tissue biotinylation was assessed using histochemistry, as described in Materials and Methods. Biotinylated tissue was revealed with omission of the avidin reagent (A) and in the presence of avidin Vectastain-ABC reagent (B). Original magnification: ×100.

The biotinylation protocol that was applied is summarized in Figure 1. The biotinylation protocol was applied to 4 classical HL samples (all samples being nodular sclerosis HL) and 3 lymph nodes with reactive lymphoid hyperplasia (RLH). All clinico-pathological characteristics are described in Table 1 and Table 2. Biotinylation was carried out to allow subsequent purification of accessible proteins. In order to assess adequacy of the biotin labelling, a representative sample of each biotinylated tissue was analysed by histochemistry with avidin-peroxidase conjugates (Figure 2). A strong staining was found in the stroma and at the cell surface in biotinylated tissues (Figure 2B). Negative control experiments in which tissues were soaked in PBS did not show any reactivity.

**Table 1:** Clinico-pathological characteristics of the Reactive Lymphoid Hyperplasia (RLH) analysed in the study

									*Intensity of immunostaining*		
***n°***	**age**	**sex**	**size (cm)**	**Localisation**	**subtype**	**CD30**	**CD15**	**EBV**	**Versican**	**Fibulin-1**	**Periostin**

RLH1	47	F	0,7	inguinal	benign lymph node	/	/	/	-	-/+	-/+

RLH2	68	M	1,3	inguinal	benign lymph node	/	/	/	-	-/+	+

RLH3	87	M	0,9	internal mammary chain	benign lymph node	-	/	/	-	-	-/+

**RLH4**	**59**	**M**	**2**	**axillary**	**follicular hyperplasia**	**+/-**	-	-	-	**-/+**	-/+*

RLH5	38	M	1,3	cervical	follicular hyperplasia	/	/	/	-	-	-/+

RLH6	24	M	1,8	axillary	follicular hyperplasia	+/-	/	-	-	-	+

RLH7	62	F	0,7	axillary	follicular hyperplasia	/	/	/	-	-	+

RLH8	22	M	1,2	spinal	follicular hyperplasia	/	/	/	-	-	+

**RLH9**	**22**	**M**	**1,9**	**submaxillary**	**mixed lymphoid hyperplasia**	-	-	**/**	-	**-/+**	-

**RLH10**	**28**	**M**	**1,9**	**cervical**	**mixed lymphoid hyperplasia**	**/**	**/**	-	**+**	**-/+**	**++**

RLH11	46	F	1,7	inguinal	mixed lymphoid hyperplasia	-	/	-	-/+	-/+	++

RLH12	38	F	1,3	subdigastric	mixed lymphoid hyperplasia	/	/	/	-	+	++

RLH13	39	F	0,9	supraclavicular	mixed lymphoid hyperplasia	+/-	/	/	-/+	-/+	++

RLH14	24	F	0,5	cervical	sarcoidosis	/	/	/	-	-/+	+

RLH15	57	M	1,5	mediastinal	sarcoidosis	/	/	/	-	-	+

Scoring of the intensity of the staining for fibulin-1, periostin and versican was performed as described in the "Methods" section. Cases in bold were analysed by mass spectrometry (see Additional File 2). The asterisk indicates that the protein was also detected by mass spectrometry in the same sample.

**Table 2:** Clinico-pathological characteristics of the nodular sclerosis Hodgkin Lymphoma (HL) analysed in the study

								*Intensity of immunostaining*		
***n°***	**age**	**sex**	**size (cm)**	**nature of lymph node**	**CD30**	**CD15**	**EBV**	**Versican**	**Fibulin-1**	**Periostin**

**HL1**	**19**	**M**	**1,3**	**cervical**	**+**	**+**	-	**+++**	**+++**	++*

**HL2**	**45**	**M**	**1,7**	**cervical**	**+**	**+**	**+**	++*	**+**	+++*

**HL3**	**21**	**F**	**2,6**	**axillary**	**+**	**+**	-	**-/+**	++*	++*

**HL4**	**27**	**M**	**2,2**	**cervical**	**+**	**+/-**	-	++*	++*	+++*

HL5	17	F	2,4	cervical	+	+	-	+++	+	++

HL6	16	F	1,6	jugular	+	-	-	+++	+++	+++

HL7	21	M	1,5	supraclavicular	+	-	-	+++	+++	+++

HL8	27	M	3,8	axillary	+/-	+/-	-	+++	+++	+++

HL9	29	M	1	cervical	+	+	-	+++	+++	+++

HL10	31	M	1,5	mediastinal	+	+	-	++	++	+++

HL11	19	F	2,5	cervical	+	+/-	-	++	++	+++

HL12	33	M	2,1	cervical	+	+/-	+	+++	+++	+++

HL13	37	F	2,5	supraclavicular	+	+/-	-	++	++	+++

HL14	22	F	0,8	cervical	+	+/-	-	+	-/+	+++

HL15	20	M	3,1	cervical	+	+/-	+	+	-	++

HL16	54	M	1	cervical	+	+	+	-/+	-	++

HL17	33	F	1	supraclavicular	+	+	-	+++	++	++

Scoring of the intensity of the staining for fibulin-1, periostin and versican was performed as described in the "Methods" section. Cases in bold were analysed by mass spectrometry (see Additional File 2). The asterisk indicates that the protein was also detected by mass spectrometry in the same sample.

### Mass spectrometry analyses

Expression profiles of biotinylated, accessible proteins were determined using the Multidimensional Protein Identification Technology (MudPIT) technique, based on 2-dimensional separation of tryptic peptide digest using nanoflow liquid chromatography coupled to electrospray tandem mass spectrometry. Each run consisted in the complete analysis in mass spectrometry of one sample.

Considering these 7 2D-LC runs, 551 ± 35 proteins were identified in each sample (mean ± SEM). Pie-charts representing the distribution of identified proteins among RLH and HL are shown in Additional File 1. Our comparative analysis of RLH versus HL nodal tissues identified several proteins known to be preferentially localized at the cell membrane or in the extracellular compartment (almost 30%). Of particular interest are those proteins overexpressed in HL. Table 3 shows a selection of 9 accessible proteins identified from biotinylated samples (see Additional File 2 for the full protein list). This selection of relevant proteins is based 1) on the regulation of the proteins (up- or down-regulation) and 2) on the actual presence of the protein either in the membrane fraction or in the extracellular space. Regulation was assessed by both "on-off" and spectral counting methods: for each analysis, a given protein was present or absent ("on" or "off"). Whenever present, we used the associated spectral counting (i.e. the number of redundant spectra of the same peptide) as a quantification mean [16], in order to have an idea of the relative protein abundance in the samples. Spectral counts for each protein are reported in Additional File 2.

**Table 3:** Selection of biotinylated proteins identified in reactive lymphoid hyperplasia (RLH, n = 3) and Hodgkin lymphoma (HL, n = 4)

Swiss Prot #	Protein Name	RLH (/3)	*SC*	HL (/4)	*SC*	Location	Biological Process
P23142	**Fibulin 1**	**0**	*0*	**2**	*3*	EC	Cell growth and/or maintenance

P13611	**Versican**	**0**	*0*	**2**	*4*	EC	Cell growth and/or maintenance

P00488	Coagulation factor XIII A1	**0**	*0*	**3**	*5*	EC, C, N	Metabolism, Transferase activity

P15531	Nucleoside diphosphate kinase A	**1**	*3*	**3**	*7*	C, N, EC, M	Metabolism; Energy pathways

P11678	Eosinophil peroxidase	**0**	*0*	**3**	*11*	C, E	Immune response

P02790	Hemopexin	**0**	*0*	**4**	*17*	E	Transport

Q15063	**Periostin, Osteoblast Specific Factor-2**	**1**	*3*	**4**	*51*	EC	Cell communication; Signal transduction

P05109	**S100 Calcium binding protein A8**	**1**	*2*	**4**	*16*	C, EC, PM	Cell communication; Signal transduction

O43399	Tumor protein D52-L2	**1**	*3*	**4**	*10*	UK	Unknown

The proteins are identified from the SwissProt database with Mascot. The numbers in bold indicate in how many runs each protein was detected. SC: Spectral counting (italicized numbers represent the number of redundant spectra identifying the same protein or group of protein). Function and location of the proteins is determined using the Human Protein Reference Database (http://www.hprd.org). C: cytoplasm, E: endosome, EC: extracellular, G: Golgi, M: mitochondrion, N: nuclear, PM: plasma membrane, UK: unknown.

Finally, in order to assess the specificity of the streptavidin column, non-biotinylated lymphoma samples were also processed and analysed by MS. In this latter case, the recovery after elution was negligible (~40X less than the average recovery from biotinylated tissues, 100 mg starting material in both conditions), and the first ten proteins identified were cytokeratins (most probably "contaminating" proteins that stuck to the streptavidin beads), showing unambiguously the specificity of our approach.

We turned our interest onto versican, fibulin-1, periostin and S100A8 proteins, because no direct relationships with HL were previously established in the literature for these proteins. Versican was identified in only two HL (out of 4), but we had learned from previous experiments that identification of peptides from this protein in complex mixtures may be difficult [15]. Therefore, we suspected that versican could be expressed in a higher proportion of diseased tissues (the same assumption applied to fibulin-1, known to interact with the versican). Since versican and fibulin peptides are potentially difficult to detect in complex mixtures using mass spectrometry (because they are large, heavily glycosylated proteins), and because of their implication in cancer, we decided to further validate these two ECM proteins using IHC. More evidently, two other markers were clearly overexpressed in HL compared to RLH: periostin and S100A8.

### Validation of the potential targets

Three proteins, namely versican, fibulin-1, and periostin are extracellular matrix proteins.

#### Versican

**Figure 3:**
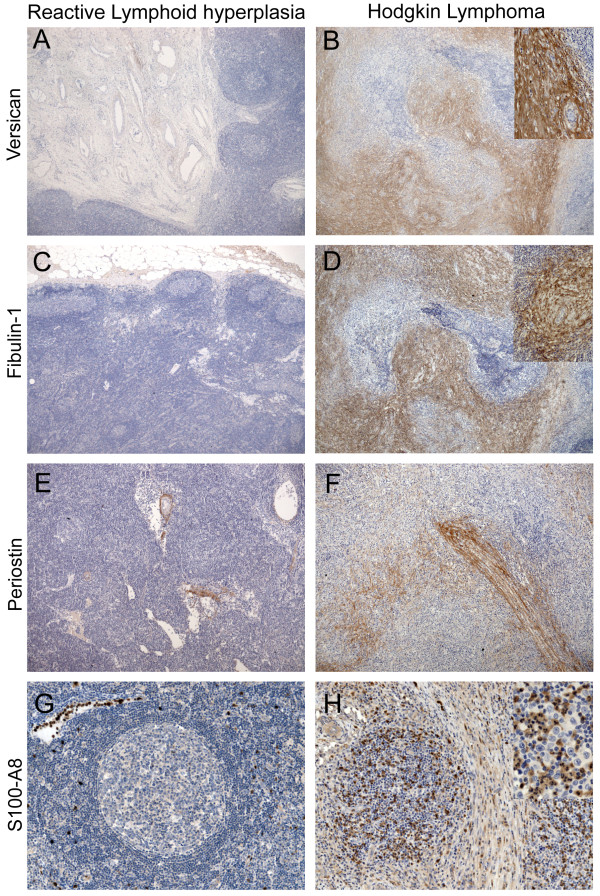
**Validation by *in situ *expression analyses**. Representative examples of versican (A and B), fibulin-1 (C and D), periostin (E and F) and S100-A8 (G and H) expression in non-tumoural human reactive lymph nodes (A, C, E and G) and Hodgkin lymphoma (B, D, F and H), as assessed by immunohistochemistry. Paraffin sections of human lymph nodes tissues were subjected to immunoperoxidase staining, as described in Methods. Original magnification: ×50; insets (B, D and H): ×100.

Versican, a large chondroitin-sulfate proteoglycan, mainly secreted by stromal cells in the ECM, is a recognized cell adhesion and motility modulator that may facilitate tumour cell invasion and metastasis [17]. While versican expression in reactive lymph nodes was generally absent (Figure 3A), moderate to strong immunoreactivity was observed in the stromal compartment of 13 out of the 17 HL tissues analysed. Fibrous bands of stroma were strongly stained (Figure 3B), and strong perivascular staining was also observed in some HL cases.

#### Fibulin-1

Proteins from the fibulin family participate in diverse biological processes, including development, cancer, and wound repair [18]. While in RLH, the antibody against fibulin-1 produced only mild and focal staining of the lymph node capsula, a strong staining of fibrous bands and capsula was commonly observed in HL (12 out of 17 cases, Figure 3D). Some perivascular staining was also present in HL.

#### Periostin

Periostin is a soluble heparin-binding N-glycosylated ECM-associated protein that is thought to contribute to cell adhesion and motility [19]. The antibody produced weak to moderate reactivity in the capsula in reactive lymph nodes and some perivascular staining (11 out of 15 RLH, Figure 3E). In HL, moderate to very strong extracellular expression in capsula and fibrous bands was observed (Figure 3F) in all cases (17 out of 17).

#### S100A8

Also known as Myeloid-Related Protein 8, this small intracellular calcium-binding protein has also been reported to be secreted, as well as expressed in the ECM of prostate cancer [20]. We found that this protein was also expressed in some parts of the ECM in both reactive and cancer lymph nodes (Figure 3G and 3H). The protein was found to be expressed by stromal cells, but also by histiocytic/monocytic cells, as well as eosinophils and neutrophils.

**Figure 4:**
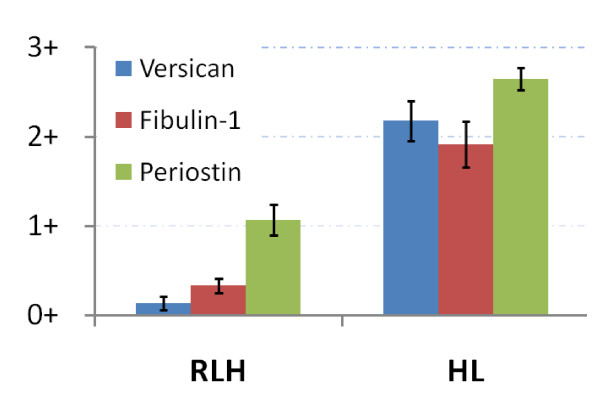
**Relative expression of three extracellular proteins**, Versican, Fibulin-1, and Periostin, as determined by IHC in RLH (n = 15) and HL (n = 17). Bargraphs represent the mean scoring (0 to 3+, determined as described in the "Material and Methods" section) from Tables 1 and 2.

Overall, overexpression in HL was clearly evident for versican, fibulin-1 and periostin (Figure 4), with a globally consistent staining pattern for each case (Table 2). Relationships between mass spectrometry and IHC for these 3 differentially expressed proteins are summarized in Additional File 3.

## Discussion

Identification of stromal proteins specifically involved in cancer is of considerable interest [21], since the "reactive stroma" of desmoplastic reactions is thought to create a permissive and supportive environment contributing to cancer progression [22]. Moreover, stromal proteins that are selectively expressed in cancer tissues are prime candidates for tumour targeting strategies because (i) they are expected to be more accessible than intracellular proteins, (ii) they are often present in high quantities and iii) tumour ECM components are generally more stable than tumour-associated cell surface antigens [23]. Considering that ~30% of patients experience relapse and die of complications due to progressive disease and/or treatment, the identification of proteins from HL stroma could not only provide the basis for a better understanding of the disease, but also lead to the discovery of new diagnostic and therapeutic markers allowing earlier detection and treatment of relapsed and refractory lymphoma by *e.g. *chemoprevention [24] or antibody based anti-cancer treatments [25].

The clinical value of antibody-based targeted therapies (targeting specifically the stroma) in HL patient has been elegantly demonstrated in a recent study using a monoclonal antibody directed against the EDB alternative spliced variant of fibronectin, known as L19 antibody [26]. In light of these promising results, it is clear that the discovery of novel accessible biomarkers in HL is a necessary step to develop a large panel of targeting-based therapies. Using a recently described chemical proteomic method [15], we have identified potentially accessible proteins that are of special interest for imaging technologies and targeted therapies in the Hodgkin's disease. Our method led to the identification of a total of 1431 proteins in HL and RLH. We used mass spectrometry as a screening tool, and only one two dimensional liquid chromatography run was performed per sample. Analytical completeness was certainly not reasonably achievable with one single 2D-LC run [27], but was not absolutely necessary to unravel new potential biomarkers (the assumption was made that a single run was likely to be sufficient to detect the most abundant proteins, and the quantitative aspect, despite analytical completeness issues, was still helpful for biomarker selection). IHC was the mandatory step to validate these potential biomarkers. Data obtained from mass spectrometry and IHC experiments on identical samples are most of the time consistent, but some discrepancies do exist (see Additional File 3). These differences can be explained by i) analytical completeness of the mass spectrometry data as stated above, ii) heterogeneity in the sample (one half for mass spectrometry processing, one half for IHC experiments) and iii) the specificity and sensitivity of antibodies used in IHC experiments. Our mass spectrometry methodology and IHC are therefore rather complementary techniques, and it can be foreseen that IHC results would more likely compare to mass spectrometry experiment such as MRM (Multiple Reaction Monitoring) that could look specifically for versican, fibulin-1 and periostin in each sample.

The complete list of identified proteins included extracellular and plasma membrane proteins, but also include a non-negligible fraction of intracellular proteins. The reasons for this observation have already been discussed elsewhere [15,28]. Focusing on ECM proteins, we identified several proteins already known to be associated with human HL. These proteins were consequently not further validated by immunohistochemistry. For instance, hemopexin protein was found elevated in the serum of patients with HL [29]; eosinophil peroxidase has already been used for radio-immunodetection of Hodgkin's disease [30]; coagulation factor XIII was found to be expressed by macrophages and involved in the stabilization of fibrin deposits in the tumour stroma of HL samples [31]; and nucleoside diphosphate kinase A expression was found to be a prognostic factor for classical HL [32].

We also identified proteins without direct relationship with HL demonstrated in the literature. Among these proteins, we showed that the extracellular proteins versican, fibulin-1 and periostin were actually overexpressed in the extracellular matrix after immunohistochemical analyses.

The staining pattern of expression was somewhat similar for the 3 proteins in most HL samples tested. This result is interesting, since the three proteins are expressed by different cells: versican is known to be expressed by fibroblasts and only rarely by cancer cells, fibulin-1 was shown to be expressed mainly by cancer cells and only by some fibroblasts [33], and periostin is expressed by both fibroblasts and cancer cells. Moreover the staining was mainly specific to fibrosis. While only scarce information is available about versican in fibrosis, except in the lung [34] (where it may influence early repair processes), fibulin-1 deposits were already reported in rat liver fibrosis [35]. Periostin, by altering the deposition and attachment of collagen, is a critical regulator of fibrosis [36].

Among these three potential biomarkers, two of them, namely versican and fibulin-1, were almost absent in RLH. Although periostin displayed a very strong expression in HL, this protein was indeed found expressed at lower levels in RLH. It would be of interest to assess the serum concentrations of each of these ECM proteins in patients with HL in order to determine how valuable seric dosage of these proteins may be for diagnostic purposes.

S100A8 was expressed in the ECM of both RLH and HL. The protein was found to be expressed in cells of the immune system (e.g. monocytes, eosinophils, neutrophils). Since these cells are typically recruited in HL lesions, the increased expression of this protein in this diseased state was expected. Results of mass spectrometry were anyway confirmed for the four proteins tested in IHC.

## Conclusion

To summarize, we identified several proteins overexpressed in the ECM of HL. The potential of these proteins for the accurate diagnosis and staging of HL deserves further investigations.

## Methods

### Tissue harvesting

Pieces of fresh human lymph nodes biopsies obtained from the Pathology department were immediately sliced and soaked into freshly prepared EZ-link Sulfo NHS-SS biotin (1 mg.ml^-1^, Pierce, Erembodegem-Aalst, Belgium) for 20 min in PBS (pH 7.4) as previously described [15]. Tissue samples were then snap-frozen in liquid nitrogen, except for a tiny portion of each sample that was directly immersed in formalin and then processed for further histological and histochemical investigations. Additional tissue samples not included in the biotinylation procedure were routinely processed for histopathological diagnosis. Controls included adjacent tissue slices incubated in PBS. Formalin-fixed, paraffin-embedded (FFPE) blocks of additional cases of nodular sclerosis HL (n = 13) and various kinds of RLH (n = 12) were retrieved from the files of the Pathology Department of the University Hospital of Liège. For all cases, diagnostics were made according to WHO classification criteria [1]. Clinico-pathological data are reported in Tables 1 and 2. All cases were reviewed by a haematopathologist (LdL). The Ethics Committee of the University Hospital of Liege reviewed and approved the specific protocol used in this study.

### Histochemistry and immunohistochemistry

Tissue penetration of the biotinylation reagent and efficiency of labelling were verified in FFPE lymph nodes tissue sections. Slides were incubated with avidin-peroxidase conjugates with the use of the Vectastain ABC kit (Vector Labortories, Burlingame, CA, USA), according to the manufacturer's instructions. Immunohistochemical validation experiments were performed as previously described [37]. For the immunohistochemical detection of versican, antigen retrieval was performed by incubating the slides with chondroitinase (Sigma-Aldrich, Bornem, Belgium). Anti-versican (clone 12C5) was developed by R. Asher, and obtained from the Developmental Studies Hybridoma Bank (University of Iowa, Department of Biological Sciences, Iowa City). This antibody was applied onto the sections at a dilution of 1:200. Anti-Fibulin-1 is an affinity isolated rabbit antibody purchased from Sigma. The immunoaffinity-purified rabbit polyclonal anti-periostin antibody was purchased from Biovendor (Heidelberg, Germany). S100-A8 (Calgranulin A) is an affinity purified goat polyclonal purchased from Santa-Cruz (Heidelberg, Germany). Control experiments included omission of the primary antibody in the procedure. Scoring of the intensity of the staining was performed as described previously [37], according to an arbitrary scale with steps of -, -/+, +, ++, and +++, where "-" was considered to be no detectable staining, "-/+" consisted in mild, focal staining, "+" was considered as weak positive staining, "++" represented moderate staining, and "+++" was considered to be strong staining.

### Sample processing

Processing was performed as previously described [15], with minor modifications. Briefly, pulverization of frozen biotinylated tissues was performed using a Mikro-Dismembrator U (Braun Biotech, Melsungen, Germany) and generated tissue powder. Approximately 100 mg of tissue powder was resuspended first in a PBS buffer containing a protease inhibitor cocktail (Halt™, Pierce). Homogenates were sonicated (2 × 30") with a 2 mm microprobe and soluble proteins were subjected to a preclearing step consisting in human serum albumin (HSA) and immunoglobulins (IgGs) depletion (Qproteome HSA and IgGs Removal Kit, Quiagen, Hilden, Germany). This step was included to limit the number of HSA and IgGs peptides detected by mass spectrometry (MS). Insoluble pellet was resuspended in RIPA buffer, and lysates were sonicated (2 × 30"), centrifuged, and further depleted in HSA and IgGs. The insoluble pellet was finally dissolved in 2% SDS. HSA- and IgGs-depleted proteins fraction and SDS-solubilized proteins were pooled and boiled for 5 minutes. Efficiency of HSA and IgG depletion was checked by SDS-PAGE and coomassie blue staining. Biotinylated proteins were then captured on a streptavidin resin (100 μl per mg of total proteins, Pierce), washed in buffer A (1% NP40 and 0.1% SDS in PBS). Protein binding was allowed for 2 hours at room temperature in a rotating mixer. After thorough washes to remove abundant cytosolic proteins with high salt buffer (buffer B: 0.1% NP40, 1 M NaCl in PBS), high pH buffer (0.1 M sodium carbonate in PBS, pH 11), and PBS. The proteins were then eluted thanks to the disulfide bonds between the lysines of labelled proteins and the biotin moieties (30' incubation steps in 1% SDS, 100 mM DTT at 58°C), allowing to bypass the direct on-resin (and thus streptavidin) digestion. Proteins were alkylated with iodoacetamide during 30' in the dark. The eluted proteins were then precipitated with 15% TCA overnight. Binding of the biotinylated proteins onto the resin and washing efficiencies were checked after SDS-PAGE, either by Coomassie blue staining or by blotting for subsequent detection of biotin by streptavidin-horseradish peroxidase (data not shown).

### Mass spectrometry

Peptide separation by reverse-phase liquid chromatography was performed on an Ultimate LC system (LC Packings, now Dionex) completed by a Famos autosampler and a Swichos II Microcolumn switching device for sample clean-up, fractionation and preconcentration. Sample (5 μg in 20 μl at 0.25 μg/μl 0.1% formic acid) was first trapped on a SCX micro pre-column (500 μM internal diameter, 15 mm length, packed with MCA50 bioX-SCX 5 μm; LC Packings, now Dionex) at a flow rate of 200 nl.min^-1 ^followed by a micro pre-column cartridge (300 μM i.d., 5 mm length, packed with 5 μm C18 PepMap100; LC Packings, now Dionex). After 5 minutes, the precolumn was connected with the separating nanoflow column (75 μm i.d., 15 cm length, packed with 3 μm C18 PepMAp100; LC Packings, now Dionex) equilibrated in mobile phase A (0.1% formic acid in 2:98 of acetonitrile:degassed milliQ water). A linear elution gradient was applied with mobile phase B (0.1% formic acid in 80:20 of acetonitrile:degassed milliQ water) spanning from 10% to 40% in 95 minutes. The outlet of the LC system was directly connected to the nano electrospray source of an Esquire HCT Ultra ion trap mass spectrometer (Bruker Daltonics, Germany), controlled by Esquire Control v6.1, build 92 and Hystar v3.2, build 44 (from the Bruker Compass software bundle). Mass data acquisition was performed in the mass range of 50-2000 m/z using the standard-enhanced mode (8100 m/z per second). For each mass scan, a data-dependent scheme picked the 3 most intense doubly or triply charged ions to be selectively isolated and fragmented in the trap. The resulting fragments were analysed using the Ultra Scan mode (m/z range of 50-3000 at 26000 m/z per second). SCX-trapped peptides were stepwise eluted with 4 CH_3_COONH_4 _concentrations, each followed by the same gradient of mobile phase B.

### Data processing and mgf file generation

Raw spectra were formatted in DataAnalysis software (Bruker Daltonics, v3.4 build 192). The portions of the 4 chromatograms (resulting from the 4 elutions with 25 mM, 75 mM, 150 mM and 500 mM CH_3_COONH_4_) containing signal (i.e. with base peak chromatogram signal above 5000 arbitrary units) were processed to extract and deconvolute MS/MS spectra, without smoothing or background subtraction. A signal/noise ratio of 3 was applied to filtrate irrelevant data in the MS/MS spectra and generate the mass list. Charge deconvolution was performed on both MS and MS/MS spectra. A retention time of 2 minutes was allowed for compound elution to minimize detection redundancy of parents of identical masses and charge states. Both deconvoluted and undeconvoluted data were incorporated in the mgf file.

### Database Searching

Proteins were identified using the minimally redundant SWISS-PROT human protein database (release 54.5, 289473 sequences, SIB; Switzerland, 17741 human entries), through the MS/MS ion search algorithm of the Mascot search engine (Mascot Server v2.1.04 and Mascot Daemon v2.1.6) running on a local 4-processor computer cluster. The mass tolerances of precursor and fragmented ions were set at 0.6 and 0.3 Dalton respectively; variable modifications were carboxymethyl cysteines and oxidization of methionines. Trypsin cuts before proline were allowed, and one misscut was also allowed. The ions score cut-off was set to 30. The absolute probability (*P*) was set to 0.05 (i.e. less than 5% probability of a random match). The full protein list generated with the above-mentioned parameters contained 1431 proteins (Additional File 2).

The false-positive rate was estimated, for each sample, by dividing the number of peptides found in the randomised SwissProt database by the number of identified peptides from the normal SwissProt database, according to following formula: fp = n random/n normal, where fp is the estimated false-positive rate, n random is the number of peptides identified (queries after filtering) from the random SwissProt database, and n normal is the number of peptides identified (queries after filtering) from the normal SwissProt database. Considering the 8 samples, fp is equal to 1.94 ± 0.15% (Mean ± SEM).

## Abbreviations

ECM: extracellular matrix; MudPIT: Multidimensional protein identification technology; NHS: N-hydroxy-succinimid; RT: Room Temperature

## Competing interests

The authors declare that they have no competing interests.

## Authors' contributions

PK performed the *ex vivo *biotinylation experiments and processed the samples for mass spectrometry analysis. PK, YG, GM and EDP contributed to the mass spectrometry analyses. LdL and DW contributed to the harvesting of the samples and performed the immunohistochemical investigations. LdL provided the tissue samples and corresponding clinical information. DW, LdL, VC and PK contributed to the design of the experiments. PK, LdL and DW wrote the paper. All authors discussed the results and commented on the manuscript. All authors agreed on the final version of the manuscript.

## Supplementary Material

Additional file 1 **Distribution of proteins identified in reactive lymphoid hyperplasia (RLH) samples and Hodgkin Lymphoma (HL) samples**. Pie charts illustrating the distribution of proteins identified in reactive lymphoid hyperplasia (RLH) samples and Hodgkin Lymphoma (HL) samples. Click here for file

Additional file 2 **Proteins identified in Reactive Lymphoid Hyperplasia (RLH) and Hodgkin Lymphoma (HL)**. List of proteins identified in 3 Reactive Lymphoid Hyperplasia (RLH) and 4 Hodgkin Lymphoma (HL) by nanoLC-ESI-MS/MS analyses. Click here for file

Additional file 3 **Relationships between mass spectrometry and IHC for 4 differentially expressed proteins**. Table showing relationships between mass spectrometry and IHC for 4 differentially expressed proteins. Click here for file
