# Supplementary figures and images for: Identification of stromal proteins overexpressed in nodular sclerosis Hodgkin lymphoma
Source: Proteome Sci. 2011 Oct 5;9:63. doi: 10.1186/1477-5956-9-63 (PMC3200160; doi:10.1186/1477-5956-9-63)

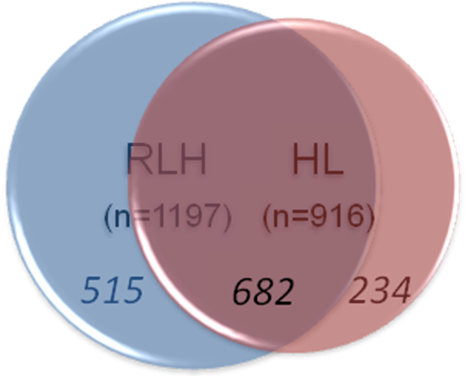

Supplement: Additional file 1 — Distribution of proteins identified in reactive lymphoid hyperplasia (RLH) samples and Hodgkin Lymphoma (HL) samples. Pie charts illustrating the distribution of proteins identified in reactive lymphoid hyperplasia (RLH) samples and Hodgkin Lymphoma (HL) samples. [file 1477-5956-9-63-S1.DOC]
