# Supplementary material for: Identification of stromal proteins overexpressed in nodular sclerosis Hodgkin lymphoma
Source: Proteome Sci. 2011 Oct 5;9:63. doi: 10.1186/1477-5956-9-63 (PMC3200160; doi:10.1186/1477-5956-9-63)
Supplement: Additional file 3 — Relationships between mass spectrometry and IHC for 4 differentially expressed proteins. Table showing relationships between mass spectrometry and IHC for 4 differentially expressed proteins. [file 1477-5956-9-63-S3.DOC]

**Additional File 3:** Relationships between mass spectrometry and IHC for 4 differentially expressed proteins.

| **Proteins** | | **MS data** | | **IHC on same cases** | **IHC on additional cases** |
| --- | --- | --- | --- | --- | --- |
| **Versican** | **RLH** | RLH4:  RLH9:  RLH10: | not found  not found  not found | negative  negative  slight positivity | Negative in 10 out of 12 cases |
|  | **HL** | HL1:  HL2:  HL3:  HL4: | not found  present  not found  present | strong positivity  strong positivity  slight positivity  strong positivity | 10 cases out of 13 with strong staining |
| **Fibulin-1** | **RLH** | RLH4:  RLH9:  RLH10: | not found  not found  not found | slight positivity  slight positivity  slight positivity | Negative in 6 out of 12 cases, slight reactivity in the 6 other cases |
|  | **HL** | HL1:  HL2:  HL3:  HL4: | not found  not found  present  present | strong positivity  slight positivity  strong positivity  strong positivity | 9 cases out of 13 with strong staining |
| **Periostin** | **RLH** | RLH4:  RLH9:  RLH10: | present  not found  not found | slight positivity  negative  strong positivity | Slight reactivity in 9 out of 12, the 3 others with strong staining |
|  | **HL** | HL1:  HL2:  HL3:  HL4: | present  present  present  present | strong positivity  strong positivity  strong positivity  strong positivity | 13 out of 13 with strong staining |

Slight positivity: +/- or +, strong staining: ++ or +++.
